# Morphometrics as an Insight Into Processes Beyond Tooth Shape Variation in a Bank Vole Population

**DOI:** 10.1371/journal.pone.0015470

**Published:** 2010-11-15

**Authors:** Ronan Ledevin, Jean-Pierre Quéré, Sabrina Renaud

**Affiliations:** 1 Paléoenvironnements et Paléobiosphère, UMR 5125 CNRS, Université Lyon 1, Villeurbanne, France; 2 Institut National de la Recherche Agronomique, UMR 1064 CBGP, Montferrier-sur-Lez cedex, France; Ecole Normale Supérieure de Lyon, France

## Abstract

Phenotype variation is a key feature in evolution, being produced by development and the target of the screening by selection. We focus here on a variable morphological feature: the third upper molar (UM3) of the bank vole, aiming at identifying the sources of this variation. Size and shape of the UM3 occlusal surface was quantified in successive samples of a bank vole population. The first source of variation was the season of trapping, due to differences in the age structure of the population in turn affecting the wear of the teeth. The second direction of variation corresponded to the occurrence, or not, of an additional triangle on the tooth. This intra-specific variation was attributed to the space available at the posterior end of the UM3, allowing or not the addition of a further triangle.This size variation triggering the shape polymorphism is not controlled by the developmental cascade along the molar row. This suggests that other sources of size variation, possibly epigenetic, might be involved. They would trigger an important shape variation as side-effect by affecting the termination of the sequential addition of triangles on the tooth.

## Introduction

Genetic networks, developmental pathways and environmental influences interact to produce, from a set of genotypes in a population, a phenotypic variation that is the target of the screening by natural selection. The variation in a population can therefore increase or impede response to selection, depending on whether or not it is correlated to the change under selection [1]. How development might channel variations from genetic or environmental origin into a preferred direction of phenotypic variation will therefore condition the capacity of a population to evolve, or evolvability [2], [3]. Analysing the patterns of phenotypic variation in a population may bring light onto the developmental constraints underlying this variation [e.g. 4], and in turn on the role that such processes might play over evolutionary time scale.

**Figure 1:**
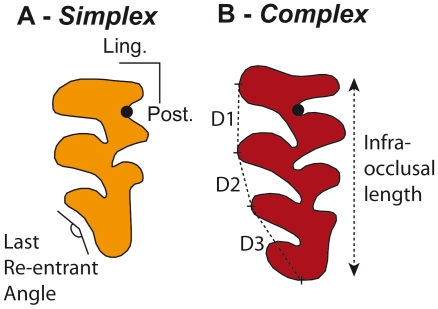
Examples of extreme morphologies of bank vole third upper molars in occlusal view. Measurements collected for this study are also presented. (A) A *simplex* molar characterised by three lingual triangles. (B) A *complex* tooth with four lingual triangles. Univariate measurements are: D1, the distance from the tips of the first to the second triangle; D2, the distance from the tips of the second to the third triangle; D3, the distance from the tip of the third triangle to the posterior end of the tooth; Infra-occlusal length, the total length of the tooth measured two millimetres under the occlusal surface on the labial side of the tooth; the last re-entrant angle, angle measuring the degree of indentation of the posterior part of the tooth. The overall shape of the tooth is quantified by the 2D outline of the occlusal surface, schematically represented here for each of the tooth. The starting point (black dot) is located at the most re-entrant point between the first and second anterior labial triangles.

The dentition of the bank vole, *Myodes glareolus* (formerly known as *Clethrionomys glareolus*) offers a challenging case to apply such quantitative methods to unravel the processes underlying phenotypic variation. This arvicoline species is known to display an important variation regarding the morphology of its third upper molar (UM3) [5], [6]. In arvicoline teeth, cusps correspond to successive triangles (Fig. 1) which number varied along the evolution of the group [7], [8]. In some species the number of cusps is fixed whereas in other a polymorphism exists regarding the number of cusps on certain molars [e.g. 9]. Exemplifying this case, the UM3 of *M. glareolus* displays from three to four triangles on its lingual side (Fig. 1A, B) whereas close relatives such as *M. rufocanus* and *M. rutilus* display three or four triangles, respectively. We therefore quantified size and shape of the UM3 in a population of *Myodes glareolus* documenting the tooth polymorphism, and using the recent advances in developmental biology of teeth [e.g. 2], [ 10], [ 11], [ 12], we inferred developmental processes underlying dental variation.

## Materials and Methods

### Study approval

This experimentation was supported by an authorization given by the French government to the co-author JPQ (n° 34–107). It allows the experimentation on living vertebrates (rodents).

### Material

A total of 103 bank voles were trapped in the same locality of Franche-Comté (surroundings of Levier, France, 6°12E, 46°58N), sampled at four successive periods of time: spring 1986 (14 specimens), autumn 1989 (27 specimens), spring 1995 (33 specimens) and autumn 1995 (29 specimens) [13]. The dry eye lens was weighted for each specimen and used as age estimator [14].

In order to compare the pattern of variation of the UM3 of *M. glareolus* with species that do not display a dental polymorphism for this tooth, 15 specimens of *M. rutilus* from Pallasjärvi (Finland, 24°11E, 68°01N) and 11 specimens of *M. rufocanus* from the Altaï (Baihaba, Xinjiang, China, 86°78E, 48°69N; [15]) were further considered. The specimens of *M. glareolus* and *M. rufocanus* are housed at the CBGP (Montpellier, France). The specimens of *M. rutilus* are part of the collection of H. Henttonen (Finnish Forest Research Institut, Vantaa, Finland).

### Size and shape univariate measurements

The length of the UM3 was measured on the labial side two millimetres below the occlusal surface in order to minimize wear effect (Fig. 1B). In order to investigate how the variation of the UM3 is conditioned by the previous teeth, the length of the first (UM1) and second (UM2) upper molars was measured as well.

The occurrence or not of an additional triangle on the lingual side of the tooth was quantified by the last re-entrant angle (Fig. 1A). To identify how much the anterior triangles influenced the polymorphism of the posterior part of the tooth, the distance from the first to the second (D1) and from the second to the third triangles (D2) were measured. The posterior part, with or without an additional triangle, was measured as the distance from the third triangle to the posterior end of the tooth (D3) (Fig. 1B).

### Outline analysis of the UM3 occlusal surface

In order to quantify the overall variation in tooth shape, the 2D outline of the UM3 occlusal surface was investigated. The elliptic Fourier transform (EFT) appears as appropriate to describe such complex shape characteristics of arvicoline teeth [16].

For each molar, 64 points at equally spaced intervals along the outline were sampled using Optimas v.6.5 and analyzed by an EFT using the EFAwin software [17]. This method is based on the separate Fourier decompositions of the incremental changes of the *x-y* coordinates as a function of the cumulative length along the outline [18]. The outline is approximated by a sum of trigonometric functions of decreasing wavelength, the harmonics. Each harmonic is weighted by four Fourier coefficients (FCs) defining an ellipse in the *x*, *y* plane: *A_n_*, *B_n_*, *C_n_* and *D_n_*. The first harmonic ellipse corresponds to the best-fitting ellipse to the outline and its area was used to standardize the FCs for size differences. The major axis of the first harmonic ellipse was taken as new *x-*axis to adjust the orientation of the outline [19]. Since the coefficients *A_1_*, *B_1_* and *C_1_* correspond to residuals after standardization [20], [21], they were not included in the subsequent statistical analysis. The coefficient *D_1_* still retains information about the elongation of the outline [22]. Hence, it was included in the statistical analyses. Ten harmonics were considered in this study, being the best compromise between the amount of measurement error and the information content of each harmonic [23]. Therefore a dataset of 37 variables (40 FCs minus *A_1_*, *B_1_* and *C_1_*) was retained for subsequent analyses.

A visualization of shape changes of the molar occlusal surface was provided by reconstruction of outlines using the inverse Fourier method [24].

### Statistical analyses

Differences in univariate parameters were tested using analyses of variance (ANOVA); their relationships with each other were investigated using linear univariate and multivariate regressions.

The set of shape variables (FCs) describing the occlusal surface of the UM3 was investigated using multivariate statistics. A Principal Component Analysis (PCA) on the 37 FCs allowed expressing the main directions of variation on synthetic shape axes. It was performed on the correlation matrix in order to balance the importance given to the FCs of the successive harmonics. These synthetic shape axes summarize the main patterns of variations, and they were investigated using the same protocols as the other univariate parameters. Two PCAs were performed, one focusing on the intra-population variation of *M. glareolus*, and the other including specimens of the related species *M. rutilus* and *M. rufocanus*.

## Results and Discussion

### Wear effect as primary source of variation of the molar occlusal surface

**Figure 2:**
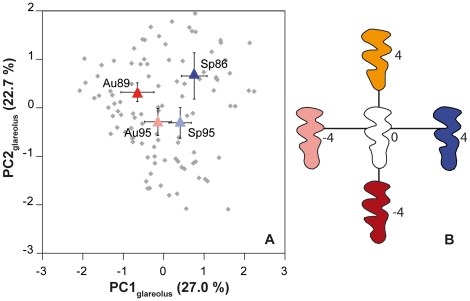
Shape variation of the third upper molar in the bank vole. (A) Shape variation of the third upper molar in the bank vole, represented on the first two principal axes of a PCA on the Fourier coefficients of the molar outline. Each grey dot corresponds to a specimen; the mean (+/− the confidence interval) of the four successive periods of trapping have been superimposed to the total variation (red squares  =  Autumn, blue circles  =  Spring). (B) Reconstructed outlines visualising shape variations along the first and second axes.

The analysis of the UM3 shape variation provided a balanced contribution on the first two principal axes (PC1  = 27.0% of total variance and PC2  = 22.7%). The first axis clearly opposes spring to autumn samples (Fig. 2A). This trend was further confirmed by two-by-two tests on scores along PC1. Both spring samples share similar PC1 scores (ANOVA: Sp86 vs. Sp95: *P*  = 0.079); both autumn samples do not differ from each other (Au89 vs. Au95: *P* = 0.079). In contrast spring and autumn populations are significantly different along PC1 (Sp86-Au89; Sp86-Au95; Sp95-Au89: *P*<0.001; Sp95-Au95: *P* = 0.022). The reconstructed outlines (Fig. 2B) show that this trend opposes teeth with a large and round forepart, characteristic of spring populations, to teeth with a straight and compressed forepart and a straight and long posterior part, typical of autumn populations.

**Figure 3:**
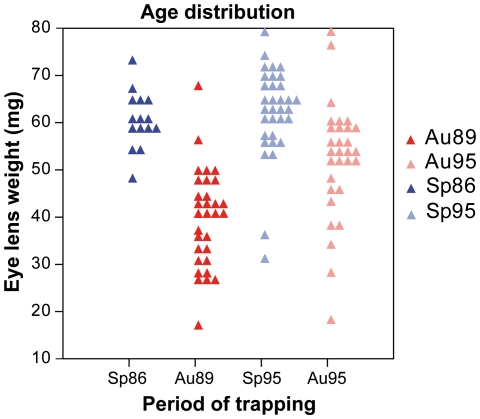
Differences in age distribution. Dot density diagram representing differences in age distribution between the successive periods of trapping. The eye lens weight (mg) was used as age estimator. Symbols correspond to the season of trapping (red squares  =  Autumn, blue circles  =  Spring).

These results are in agreement with previous data on a Finnish bank vole population that evidenced an important effect of the trapping season [6]. The reason of this effect was hypothesized to be due to different age structure in spring and autumn populations, leading to different wear stages of the tooth dominating at different time periods of the year. Progressive wear down the crown was shown to impact the shape of the occlusal surface [6] in a similar way to the one characterizing the present trend along PC1. Our data set allows testing this hypothesis in a more direct way: eye lenses were weighted in our populations and provide a direct estimate of the age of each animal [14]. This age estimator shows dramatic variations from autumn to spring (Fig. 3). Autumn populations are dominated by young animals born in spring and summer [25] whereas spring populations are exclusively composed of old, overwintered animals. The significant relationship between this age estimator and the shape of the UM3 occlusal surface, estimated by scores on PC1 (*R^2^* = 0.277, *P* = 0.001) further validates the hypothesis that population dynamics, by affecting the wear stage dominating each sample, heavily contributes to the variation in occlusal shape in this species. An additional source of difference in the degree of wear between spring and autumn samples might relate to the food consumed by the animals that supposedly varies depending on the season.

This result may appear surprising since wear has been demonstrated of little importance on 2D outline of the tooth in other rodents such as mice (e.g. [26], [4]). This discrepancy regarding sensitivity of the outline shape to wear is due to the different geometry of the cusps. The bulged cusps in murine teeth allow focusing down the crown when analyzing its 2D outline, a place affected by wear only late in life; in contrast the vertical sides of the triangles on the arvicoline teeth force to consider the shape of the occlusal surface, directly affected by wear.

This source of variance related to population dynamics may appear as both an advantage and a drawback, depending on the scopes of morphometric analysis of tooth shape. It is a drawback if trying to analyze evolutionary or biogeographic patterns, by introducing a contingent factor related to the time of trapping for each sample (e.g. [23]). It is challenging, on the other hand, by opening the way to study some aspects of population dynamics on past samples. Regarding this perspective, a year effect possibly emerges in addition to the seasonal differences, both along PC1 and PC2, opposing samples from 1986 and 1989 to samples from 1995. Especially, the morphological closeness of the two samples from 1995 challenges some further analyses. It may either be due to a genetic relatedness, sampling two directly successive generations of bank voles, and this would suggest the occurrence of a dynamics of genetic changes through time, either due to drift or selective effects. Alternatively, being so close in time, the two generations documented in Sp95 and Au95 might have experienced related environmental and/or demographic conditions during growth [27], leading to a close morphological signature.

To conclude on this first-order morphological signal, it appears as related to intra-annual, and possibly inter-annual, population dynamics. As such, it is not directly relevant either to genetic variations or developmental processes during the formation of the tooth. It rather corresponds to a superimposed signature of wear during late life that may blur other, more intrinsic sources of variations.

### Intra-population variation in UM3 shape: modulation of an unchanged developmental pathway?

Focusing on the second axis of variation (PC2) allows discarding the variation of the tooth superimposed by wear during late life. This axis opposes teeth with three lingual triangles to teeth with an additional triangle, suggesting that this axis may correspond to the polymorphism described in the bank vole [5]. We confirmed this interpretation by evidencing a correlation between scores on PC2 and the re-entrant angle on the posterior part of the tooth quantifying the occurrence of a fourth lingual indentation (*R^2^* = 0.525, *P*<0.001). This polymorphism was first described by its two end-members, the morph with three triangles being named *simplex* and the morph with four triangles *complex* [28], [5]; considering the occurrence or not of a triangle, together with this clear-cut terminology, suggested a discrete variation [29]. Our results rather show a continuous range of variation between the two characteristic end-members. They further suggest that the typical and most obvious polymorphism, corresponding to the fourth lingual indentation, may go together with other concerted changes of the occlusal surface, since the reconstructed outlines point to other morphological trends along PC2 such as a broader vs. narrower anterior part of the tooth (Fig. 2B).

Developmental data have shown that triangles in arvicoline teeth, in the same way than cusps in murine molars [30], are sequentially determined along a mesio-distal and temporal sequence. Activation-inhibition mechanisms control spacing and timing in such a sequential addition [2]. The first cusp to develop, corresponding at this ontogenetic stage to an epithelial signaling center (a.k.a enamel knot), is surrounded by an inhibitory field and the next cusp will only develop outside this field. The spacing of the cusps, here the triangles, are thus likely determined by the range of this inhibitory field; the phenotypic output on the adult tooth can be estimated as the distance between successive triangles. The first way to achieve an additional triangle on the UM3 would thus involve an alteration of the inhibitory field: the shorter the spacing between successive triangles, the more triangles are likely to develop for a tooth of equivalent size. An alternative is to vary the posterior termination of the sequential addition, without changing the spacing between triangles.

**Figure 4:**
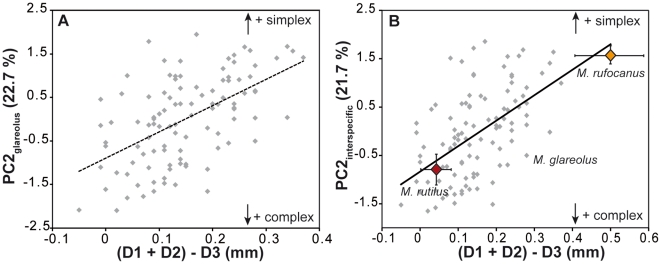
Relationship between overall shape of the tooth and local variations in tooth dimensions at the intraspecific (A) and interspecific (B) scale. The second synthetic shape axis PC2 is taken as an estimator of the *simplex-complex* variation. It is compared to a combination of the inter-triangles distances (D1+D2)−D3: the smaller (D1+D2), the shorter the spacing between the first triangles; the higher D3, the longer the posterior part of the tooth. Both mechanisms can contribute to the formation of the additional triangle typical of *complex* teeth, leading to a correlation between PC2 and (D1+D2)−D3. (A) Variation within the bank vole *M. glareolus*. (B) To include variation at a higher evolutionary scale, specimens of *M. rufocanus* (*simplex* UM3) and of the *M. rutilus* (*complex* UM3) have been added to the intra-specific variation of the bank vole. The shape axis PC2 was recalculated on the basis of the new dataset.

A change of the spacing would affect the distance between the first triangles, hence D1+D2. A change in the termination of the sequential addition would lead to a variation in the posterior part of the tooth, hence D3. Therefore, the chance to develop an additional lingual triangle (quantified by lower scores on PC2) would increase with a decrease of (D1+D2), or an increase of D3. Whatever the process, PC2 should thus be related to (D1+D2)-D3 and this is indeed the case (*R^2^* = 0.303, *P*<0.001; Fig. 4A). Yet, PC2 is not related to D1+D2 (*R^2^* = 0.003; *P* = 0.561) but is related to D3 (*R^2^* = 0.355, *P*<0.001).

**Figure 5:**
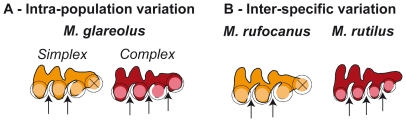
Schematic representation of the evo-devo model proposed to explain the polymorphism in the UM3 shape. The formation of each triangle is controlled by a signalling centre (colour-filled circle) surrounded by an inhibitory field (black circle); the subsequent triangle can only develop outside this inhibitory field. (A) Intra-population variation in the bank vole is not related to a change in the spacing of the triangles (hypothesized as related to a change in the dimension of the inhibitory field during the tooth development), but to the termination of the process towards the posterior end of the tooth: an additional triangle will form if enough space is available. (B) In contrast, differences between species such as *M. rufocanus* and *M. rutilus* are related to a change in the spacing of the triangles.

This suggests that the mechanism involved in the occurrence of an additional triangle is not related to a change in the spacing of the triangles, but rather to a change in the termination of the process of sequential addition of the triangles (Fig. 5A). Such a mechanism is known to produce intra-specific variation in other morphological features, e.g. the number of palatal ridges in some muroid rodents [31]. A slight change in the timing of the termination or in the size of the field where the sequential addition takes place (here the dental lamina) can easily achieve a marked phenotypic output by overriding the threshold necessary to the formation of an additional sequential feature. Interestingly, such a variation can occur without changing the basic properties of the developmental process, by slight modulations of the developmental pathway that might even be of epigenetic origin. This challenges the traditional view of the *simplex-complex* polymorphism as due to a supposed, if not identified, genetic variation [5].

### Different processes at work on different evolutionary scales?

The *simplex-complex* variation of the UM3 also occurs at a higher taxonomic level between related species. Whereas this feature is variable in the bank vole, it is fixed in other species. We wondered if this inter-specific variation occurred without change in the underlying developmental process, as in the case of the intra-specific variation within the bank vole, or if the developmental process itself could be affected at this evolutionary scale.

We thus performed another analysis of the tooth shape variation (Fig. 4B), including together with the bank vole samples a set of Northern red-backed voles (*M. rutilus*) displaying a *complex* morphology with four lingual triangles, and of Grey red-backed voles (*M. rufocanus*) displaying a *simplex* morphology (Fig. 5B). The scores of the bank voles on this new “inter-specific” PC2 are highly correlated to their scores on the former “intra-specific” PC2 (*R^2^* = 0.951, *P*<0.001) showing that direction of PC2 is comparable in the two analyses. This “inter-specific” PC2 is, as previously, related to either a shortening of the spacing between successive cusps, or a lengthening of the posterior part (correlation with (D1+D2)−D3: *R^2^* = 0.431, *P*<0.001). The balance between the two processes, however, is changed when compared with the intra-specific variation of the bank vole alone. PC2 is still related to D3 (*R^2^* = 0.329, P<0.001) due to the massive sampling of the intra-population variation of the bank vole. Newly, however, PC2 is also related to a change in the spacing of the triangles (correlation with D1+D2: *R^2^* = 0.199, *P*<0.001).

These results point to the fact that different mechanism may underlie a similar phenotypic variation, depending on the evolutionary scale considered (Fig. 5). A change in the spacing of the successive triangle is involved in the inter-specific variation that is not involved in the intra-specific variation. Changing the spacing of the triangles requires changing the intrinsic properties of the sequential development of the cusps, probably underlain by some genetic differences. The three species of bank voles diverged a few million years ago (∼4 My for *M. rufocanus* and ∼3 My for *M. glareolus – M. rutilus*; [32], [33]), a time span long enough to accumulate such differences in the basic properties of the sequential development of the triangles. Since the oldest species, *M. rufocanus*, displaying a *simplex* morphology whereas the two most recent species display polymorphic (*M. glareolus*) or *complex* teeth (*M. rutilus*), this lineage might exemplify a trend towards an increase of complexity of the tooth pattern [8].

In contrast, the intra-specific variation in the bank vole seems rather to depend on modulation of an unchanged developmental process. The process could be related either to a postponing of the termination of the sequential addition of the cusps, or a more elongated dental lamina without changes in the developmental timing. Both are hardly distinguishable based on their phenotypic output on the formed tooth: they both correspond to a slightly longer posterior part of the UM3 (here quantified by D3). We attempted to investigate, as the next step, the possible cause of this variation triggering the *simplex-complex* polymorphism, by investigating patterns of UM3 size variation.

### UM3 size weakly canalised by the cascade along the tooth row

Teeth do not develop independently along the tooth row. Another process of sequential addition, controlled by a balance of activation and inhibition, is at work in the development of the successive molars. Developmental evidences on the lower molar row of murine rodents suggested a cascade where the first molar inhibits the second and the second the third, the activation-inhibition balance remaining stable at each step of the process. This leads to a cascade mainly controlled by the size of the first molar [12]. This cascade seems to control molar proportions in many taxa [34], although arvicantine rodents emerge as an exception. The pattern of sequential addition in this group seems to be modified from the second to the third tooth, leading to a third molar both controlled by the first and second teeth [35].

**Figure 6:**
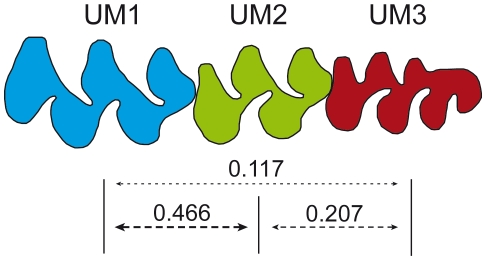
Relationships between upper molar size. Schematic representation of the relationships between the size of the molars along the upper molar row of the bank vole. Relationships are represented as arrows, the thickness of the arrow being proportional to the strength of the relationship indicated by *R^2^* coefficients.

If these models of development can be extrapolated to the upper molar row, the crucial variation at the posterior end of the UM3 in the bank voles might be triggered by changes much earlier in the development and involving the size of the first and/or second molars. We thus tested this hypothesis by considering how the sizes of the different teeth were related (Fig. 6).

As expected in both models, the sizes of the three teeth are correlated with each other (*P*<0.001), suggesting that the cascade model of development evidenced on lower molars may be valid for the upper molar row as well. The strength of the relationship, however, varies greatly depending on the teeth. The first and second molars appear to be highly correlated, their relationship explaining almost 50% of the UM2 variation (UM1-UM2: *R^2^* = 0.466). In contrast, the size of the UM3 is much less strongly related to the other teeth; its relationship with UM2 explains only about 20% of its variation and even less regarding its relationship with UM1 (UM2-UM3: *R^2^* = 0.207; UM1-UM3: *R^2^* = 0.117). This weak relationship to the UM1 is confirmed by a multiple regression model showing a significant relationship of UM3 size with UM2 but not with UM1 (UM3 vs. UM1 and UM2: *R^2^* = 0.209, UM1: *P* = 0.662, UM2: *P* = 0.001). This is in agreement with the cascade model specific to arvicoline rodents [35] but this raises a few comments.

First, the model was based on inter-specific relationships and we evidence here that a similar process seems at work on an intra-specific scale. The percentage of variance explained, however, is much less for intra- than inter-specific variation: more that 90% of UM3 size variation is explained by the inter-teeth cascade at the inter-specific scale [35], whereas only 20% are explained regarding intra-specific variation in the bank vole. This suggests that considering inter-specific variation probably buffers other sources of variation occurring at an intra-specific scale.

Second, both cascade models were developed based on data on the lower molar row. We provide here evidences that similar processes may be at work on the upper molar row. This undermines the argument for a specific model for arvicolines: the predominant role of the second molar was attributed to the hypermolarization of the first lower molar [30], [36]. Our results rather suggest that the pivotal role of the UM2 in the cascade might characterise the developmental pathway of arvicolines independently of the hypermolarisation of the first lower molars. By conferring a certain independence for the evolution of the first molar, this change in the cascade might have been the key, rather than the consequence, of the hypermolarisation process.

Regarding the mechanisms controlling the size of the UM3, our results provide balanced evidences. The size of the UM3 is partly controlled by a developmental cascade along the tooth row, but this cascade explains only a minor proportion of the UM3 size variation, and hence presumably not the variation triggering the *simplex-complex* polymorphism. This evidences a lower canalisation of the third molar compared to the first teeth of the molar row, a result repeatedly found in rodents, arvicolines [37] as well as murines [4], and up to large mammals [38]. The position of the third molars at the end of the molar row makes it prone to cumulate any changes, genetic or epigenetic, occurring former in the development of the molar row. Developing later, it might also vary in response to other cues than the former teeth, including non-genetic influences. The marked phenotypic polymorphism might hence be the by-product of another variation, determining the size of the molar row and/or the timing of termination of the sequential addition of cusps. The range of candidate factors includes epigenetic factors, such as maternal health, that have been documented to affect tooth size [39], [40]. Maternal health and body size may in turn be affected by many factors according to the complex bank vole ecology: variations in abiotic environment and nutritional quality [41], population dynamics and density-dependent factors [42], [43]. Such a complex and subtle interplay of mechanisms underlying the variation in the shape of the third upper molar of the bank vole may explain why clear geographic pattern emerges only as secondary signal of variation [23]. It also provides a challenging scenario explaining the evolvability of this tooth through evolution, although the developmental processes at work may vary depending on the evolutionary scale considered.

### Concluding remarks

Main directions of phenotypic variance are suggested to constitute “lines of least resistance” to evolution [44], by representing morphologies more frequently produced in a population due to genetic or developmental factors, and hence more easy to screen by selection or to randomly sample by drift. Yet, their meaning for long-term evolution depends on the degree of heritability of the phenotypic variation.

The present study provides contrasted evidences in this respect. The first direction of variance corresponds to the degree of wear of the tooth, and hence appears as a signal superimposed to the intrinsic variance by contingent factors during the late life of the animals. These factors are likely conditioned by local life-history traits.

The second direction of intra-population variance seems to be produced by variation during the development. Yet, the developmental process, corresponding to a sequential addition of triangles, is not modified; rather, the variation in tooth shape seems to be due to variations in the termination of the process. The shape polymorphism would thus occur as a side-effect of variation in the posterior elongation of the tooth. Such a feature may be heritable, and possibly itself under selective pressure, or be of epigenetic origin and related to life-history traits. The frequent interpretation of dental variation as a result of selection for efficient food processing (e.g. [45], [9]) would be dramatically challenged if the tooth polymorphism would be of epigenetic origin.

Yet, the processes involved seem to be different at a higher evolutionary level. Inter-specific differences in tooth shape are related to a change in developmental process, namely in the spacing between successive triangles. Such a modification is likely heritable and supports adaptive scenarios of dental shape evolution.

The combination of several processes generating a similar direction of morphological variation might be a key of the remarkable evolvability of tooth shape among population and species in arvicoline rodents [8].
